# Protocol for evaluation of neurotrophic strategies in Parkinson’s disease-related dopaminergic and sympathetic neurons *in vitro*

**DOI:** 10.14440/jbm.2016.124

**Published:** 2016-07-25

**Authors:** Shane V. Hegarty, Aideen M. Sullivan, Gerard W. O’Keeffe

**Affiliations:** ^1^Department of Anatomy and Neuroscience, Biosciences Institute, University College Cork, Cork, Ireland; ^2^The Irish Centre for Fetal and Neonatal Translational Research (INFANT), Cork University Maternity Hospital, Cork, Ireland

**Keywords:** neurite growth analysis, dopaminergic neuron, Parkinson’s disease, neurotrophic therapy, sympathetic neuron

## Abstract

Parkinson’s disease (PD) is a neurodegenerative disease that is characterized by motor and non-motor symptoms which result from the progressive degeneration of nigrostriatal ventral midbrain (VM) dopaminergic (DA) neurons, as well as peripheral sympathetic neurons. PD is incurable, with current therapeutic strategies providing symptomatic relief. Neurotrophic factor (NTF) therapy has the potential to protect degenerating neurons in PD. However, there has been limited success in PD clinical trials due to neurotrophic strategies that are invasive, inefficient in delivering sustained neurotrophic support, do not protect all degenerating neurons and may have a compromised mechanism of action in the PD brain. Therefore, while neurotrophic therapy remains a promising disease-modifying approach for PD, it is important to identify novel neurotrophic strategies that can protect all neurons affected by PD. To address this need, we report an integrated approach for pre-clinical evaluation of potential neurotrophic strategies, *e.g.*, pharmacological agents (*e.g.*, drugs/small molecules), signaling proteins (*e.g.*, morphogens) and/or genetic (gene/mRNA) modifications, in cellular models of the neuronal populations that are affected by PD. Herein, we describe, in detail, an *in vitro* protocol that allows a step-wise evaluation of the efficacy, and mechanism(s) of action, of novel neurotrophic strategies in VM DA neurons and sympathetic neurons, following an initial evaluation in a human cell line model of these cells, SH-SY5Y cells. The protocol uses the induction of neurite growth as the primary measure of neurotrophic action. Indeed, the neuro-protection/-restoration of PD-affected axons is widely thought to be an appropriate target for effective therapeutic intervention in PD.

## BACKGROUND

Parkinson’s disease (PD) is the second most common neurodegenerative disease that affects 1–2% of people over the age of 65, and this incidence significantly increases with age [1]. In PD, the progressive degeneration of ventral midbrain (VM) dopaminergic (DA) neurons of the nigrostriatal pathway results in reduced DA neurotransmission in the striatum, leading to motor dysfunction. PD is thus largely characterized as a motor disorder, but also presents with a variety of accompanying non-motor symptoms [2,3]. Another defining feature of PD is the pathological accumulation of intraneuronal Lewy bodies, consisting predominantly of α-synuclein, that are found throughout the central, peripheral, and autonomic nervous systems [4-6]. In parallel to the striatal DA denervation [7], there is also heterogeneous peripheral denervation [8-10], such as sympathetic denervation of the left ventricular myocardium that leads to hypotension [11]. Despite decades of research, current PD treatments are symptomatic and mainly involve dopamine-replacement strategies. However, these treatments wear off over time and do not slow, prevent or reverse the degeneration of neurons affected by PD [12].

At present, neurotrophic factor (NTF) therapy is one of the most promising disease-modifying therapies for PD. NTFs are critical for the survival and growth of neurons in the nervous system. A number of neurotrophic factors have been shown to promote the survival and growth of VM DA neurons *in vitro* and *in vivo*, many of which are members of the transforming growth factor β superfamily including glial cell line-derived neurotrophic factor (GDNF), neurturin (NTN) and growth/differentiation factor 5 (GDF5) [13-15]. The application of NTFs to the striatum and/or VM thus has the potential to protect degenerating VM DA neurons in PD [14]. However, this approach has had limited success in clinical trials to date [14-16]. One potential explanation for ineffectiveness of GDNF and NTN NTFs, used in these clinical trials, is the suggestion that these NTFs may not be capable of signaling in the PD brain. Indeed, α-synuclein has been shown to downregulate expression of the GDNF/NTN receptor, RET, in the α-synuclein rat model of PD, in which GDNF has no neurotrophic effects [17,18]. Indeed, authors of the most recent NTF PD clinical trial suggested that “better results might be achieved with other trophic factors that are not RET dependent” [16]. In addition to this, localised administration of NTF ligands in PD will not protect the other peripheral/autonomic neurons affected by PD. Furthermore, such intracerebral administration is invasive, and inefficient in its delivery of sustained neurotrophic support [15]. Therefore, while neurotrophic therapy remains a promising disease-modifying approach for PD, it is important to identify novel neurotrophic strategies that may be useful in protecting all neurons affected by PD.

In order to develop novel neurotrophic strategies, we need a systematic protocol for pre-clinical evaluation of potential neurotrophic strategies in neuronal populations which are affected by PD. The mechanisms of action of any neurotrophic strategies also needs to be characterized in order to ensure that such mechanisms are capable of functioning in the PD brain before trials in humans. Herein we report an integrated approach for investigating neurotrophic efficacy in VM DA and sympathetic neurons, following initial evaluation in a human cell line model of these neuronal populations that are affected by PD. As neurotrophic factors function to promote the survival and neurite growth of neurons during development and in adulthood, this protocol focuses on the induction of neurite growth as the primary measure of neurotrophic action. Indeed, neuro-protection/-restoration of PD-affected neurites is essential to modify the progression of PD, as axonal degeneration has been reported to be the earliest pathological feature of PD, as well as an appropriate promising target for effective therapeutic intervention [19].

## MATERIALS

### Animals

Embryonic day (E) 14 embryos were removed by laparotomy from euthanized time-mated Sprague-Dawley rats. Postnatal day (P) 1 C57 mice or Sprague-Dawley rats were decapitated. All animals were provided by Biological Services Unit, University College Cork, and procedures were carried out with approval of the Animal Experimental Ethics Committee of University College Cork.

### Equipment

• Class II Microflow Biological Safety Cabinet
• Tissue Culture Incubator (ThermoForma Series II, Thermo Electron Corporation)
• 15 ml conical centrifuge tubes (Corning)
• 0.5/1.5 ml Eppendorfs (Greiner)
• T25 flasks (Greiner)
• 24-well and 6-well plate tissue culture plates (Sarstedt)
• Plugged and unplugged flame-polished glass Pasteur pipettes (Sarstedt)
• Haemocytometer (Marenfield Superior)
• 90 mm Petri dish (Fannin Healthcare)
• Dissection microscope (Leica Wild M8) in a fume hood (Brassaire)
• Dissecting scissors, tweezers and a curved forceps (World Precision Instruments)
• 5 ml syringe (BD PlastipakTM) and 1.10 × 30 mm needle (Sterican)
• 4 well 35 mm tissue culture dishes (Greiner)
• Centrifuge (15 ml tubes & eppendorfs)
• NeonTM Transfection System electroporator, pipette, cuvette and tips (Invitrogen)
• Microplate spectrophotometer and laboratory rocker
• Olympus IX70 inverted microscope fitted with Olympus DP70 camera (Mason Technology)
• Scaled 1 mm micrometer slide (GT Vision)
• AnalysisD, ImageJ, GraphPad Prism and Matlab software

### Reagents

• Human SH-SY5Y Neuroblastoma Cell Line (Sigma, cat. # 94030304)
• Foetal bovine serum (FBS; Sigma, cat. # F2442)
• Trypsin (Sigma, cat. # T6567)
• Dimethyl Sulfoxide (DMSO; Sigma, cat. # D2650)
• Dulbecco’s Modified Eagle Medium Nutrient Mixture F-12 (DMEM-F12; Sigma, cat. # D6421)
• L-Glutamine (Sigma, cat. # G7513)
• Penicillin/Streptomycin (Sigma, cat. # P4458)
• Hank’s Balanced Salt Solution (HBSS; with NaHCO3, without phenol red/Ca2+/Mg2+; Sigma, cat. # H6648)
• B-27 Supplement (Invitrogen, cat. # 17504044)
• N-2 Supplement (Invitrogen, cat. # 17502048)
• Poly-D lysine, poly-ornithine and laminin (Sigma, cat. # P1149, P4957, L2020)
• Nerve growth factor (NGF; R&D Systems, cat. # 256-GF-100)
• Broad_spectrum caspase inhibitor Boc_D_FMK (Calbiochem, cat. # 218745)
• Calcein-AM (Invitrogen, cat. # C1430)
• Transit 2020 Transfection Reagent (T2020) (Mirus, cat. # MIR6003)
• Phosphate-buffered saline (PBS) (without CaCl2 and MgCl2; Sigma, cat. # D5652)
• NeonTM Transfection System resuspension and electrolytic buffers (Invitrogen, cat. # MPK10025)
• Thiazolyl Blue Tetrazolium Bromide (MTT; Sigma, cat. # M5655)
• Methanol (Sigma, cat. # 322415)
• Paraformaldehyde (PFA; Sigma, cat. # P6148)
• Bovine serum albumin (BSA; Sigma, cat. # A2153)
• Mouse anti-tyrosine hydroxylase (TH; Millipore, cat. # MAB318)
• Mouse anti-β-actin (Sigma, cat. # A5441)
• Goat anti-mouse/rabbit Alexa Fluor 488/594-conjugated secondary antibodies (Invitrogen, cat. # A11001, A11005, A11008, A11037)
• 4’-6-Diamidino-2-phenylindole (DAPI; Sigma, cat. # D8417)

### Recipes

• *SH-SY5Y Media*: DMEM-F12, containing 10% FBS, 100 nM L-Glutamine, 100 U/ml Penicillin/Streptomycin
• *Differentiation Media*: DMEM-F12 supplemented with 100 nM L-Glutamine, 100 U/ml Penicillin/Streptomycin, 1% FBS and 2% B-27 Supplement
• *SCG Media*: DMEM-F12 supplemented with 100 nM L-Glutamine, 100 U/ml Penicillin/Streptomycin, 1% N-2, 2% B-27 and 10 ng/ml NGF (or 50 μM Boc_D_FMK)

## PROCEDURE

The following is a step-wise procedure designed to identify and evaluate potential neurotrophic strategies in three cell culture models. The protocol uses the induction of neurite growth as the primary measure of neurotrophic action. The human SH-SY5Y neuroblastoma cell line is a model of human adrenergic and dopaminergic neurons. SH-SY5Y cells are first used to identify, screen and evaluate potential neurotrophic strategies, *e.g.*, pharmacological agents (*e.g.*, drugs/small molecules), signaling proteins (*e.g.*, morphogens) and/or genetic (gene/mRNA) modifications. Effective neurotrophic strategies in SH-SY5Y cells are then evaluated in VM DA neurons and sympathetic neurons *in vitro*.

### Evaluation of potential neurotrophic strategies in SH-SY5Y cells

1. Cell culture of human SH-SY5Y neuroblastoma cell line
  1.1. Culture SH-SY5Y cells at a seeding density of 2 ×10^6^ cells in a T25 flask in 10 ml of SH-SY5Y Media at 37°C in a humidified atmosphere containing 5% CO_2_.
  1.2. When ~80% confluency is reached, remove media, wash cells in 1 ml of HBSS and then enzymatically dissociate in 0.2% trypsin by incubating at 370C for 5 min.
**NOTE**: At this density, ~80% confluency is reached after ~7 days *in vitro* (DIV).
  1.3. Add 1 ml of SH-SY5Y Media to neutralize trypsin, and vigorously triturate cells 3–5 times using a plugged flame-polished Pasteur pipette (while avoiding air bubbles).
  1.4. Prepare a 1:10 dilution of the cell suspension in media for cell counting with a haemocytometer. Count 5 grids and calculate total number of cells/ml: Cells/ml = (Number of Cells Counted in 5 Fields)/5 × Dilution Factor × Haemocytometer Constant
  1.5. Plate SH-SY5Y cells at a density of 50,000 cells/well (in 500 µl of media) in 24-well plate and incubate at 370C. Replace half of the media every 2–3 DIV (if no treatment).
2. Identification of viable working concentrations of potential neurotrophic agent in SH-SY5Y cells
  2.1. From 1 DIV, treat SH-SY5Y cells with a range of doses/concentrations (*e.g.*, 1, 10, 25, 50, 100, 200 µM) of chosen pharmacological agent, or signaling protein, daily for 4 DIV. Treat control cells with equivalent volume of DMSO and/or HBSS.
**NOTE**: 4 DIV is optimal time point to analyze SH-SY5Y neurite growth [20].
  2.2. To treat cells, prepare mixture of media and agent to give desired concentration in 500 µl of media/well. Replace entire media with relevant treatment media.
**Tip**: Consult literature for effective doses of chosen potential neurotrophic agent in SH-SY5Y cells, or similar cells, to inform choice of concentrations range for testing.
  2.3. At 4 DIV, assess cell viability by performing an MTT Assay as follows:
  2.4. Remove media from the wells of the 24 well plate, and incubate cells with 300 µl per well of 0.5 mg/ml MTT in HBSS for 4 h at 37°C
**NOTE**: Phase-contrast images can be taken as observational measure of cell viability. A lactate dehydrogenase assay using the media can also be used to assess toxicity.
  2.5. Carefully remove MTT solution from wells (do not disturb insoluble formazan).
  2.6. Add 100 µl of DMSO to each well to permeabilize formazan produced by the cells.
  2.7. Pipette 75 µl of colored DMSO/formazan solution into a 96 well plate, and record the absorbance (αviability) of each sample using a plate reader at A600.
3. Evaluation of neurite growth-induction ability of potential neurotrophic agent in SH-SY5Y cells
  3.1. From 1 DIV, treat SH-SY5Y cells with viable dose(s) (from Step 1.2) daily for 4 DIV.
  3.2. At 4 DIV, fix (4% PFA), block (5% BSA) and immunocytochemically stain SH-SY5Y cells for β-actin/DAPI (as per ref. [20]) to allow visualization of cell cytoskeleton.
  3.3. Alternatively, calcein-AM can be used for visualization of cell structure. To do this, add calcein-AM to culture media at a dilution of 1:1000 for 1 h before imaging.
**NOTE**: Image live cells immediately to avoid over-exposure of calcein-AM staining.
  3.4. To measure neurite growth, image 20 microscopic fields per group, selected at random, at 200 × magnification (using a 20 × objective lens). Image both β-actin- and DAPI-stained cells in each field. Systematically image similar fields in every well to minimize bias. Measure all cells in each image using stereological procedures [21].
  3.5. Open images in ImageJ and superimpose gridlines on the microscopic image. Using the ImageJ cell counter, manually count and record the number of times each neurite (β-actin- or calcein-AM-stained) intersects gridlines, and the number of cells (DAPI).
  3.6. Calculate total neurite length using the formula; NL = α × T × (π/2) where α is the number of times the neurites intersect the grid lines, and T is the distance between the gridlines on the magnified image (taking into account the 200 × magnification factor).
**NOTE**: Measure T by superimposing gridlines on 200 × image of scaled 1 mm slide. T of 25 µm is recommended for SH-SY5Y cell neurite analysis.
  3.7. Calculate neurite length per cell by diving total neurite length by number of cells within each image (to prevent differences in neurite length being due to cell number).
4. Evaluation of neurite growth-induction ability of genetic modification in SH-SY5Y cells
  4.1. To evaluate the potential neurotrophic role of a target gene/protein, and/or to verify results achieved in above pharmacological approaches, transfect SH-SY5Y cells with GFP (for visualization) and the plasmid(s) of interest (*e.g.*, over-expression, siRNA).
  4.2. For transfection, electroporate SH-SY5Y cells using the NeonTM Transfection System.
**NOTE**: Alternatively, lipo-transfection can be performed using the Transit 2020 Transfection Reagent, but yields lower transfection efficiency (see Troubleshooting).
  4.3. Prepare cell suspension (as per Step 1), and then centrifuge required volume of cells to give 200,000 cells/well at 1,100 rpm for 5 min. Wash cell pellet twice in PBS, and resuspend cells in required amount of resuspension buffer (12 µl per transfection).
  4.4. Add 0.5 µg of GFP and 1 µg of desired plasmid to the resuspended cells, and aspirate 10 µl of the cells/plasmid DNA mixture into a gold Neon Tip using the Neon Pipette.
  4.5. Place into cuvette, containing 3 ml of electrolytic buffer, in the Neon Pipette Station. Deliver electric pulse using optimized parameters: 1200 V, 20 ms width and 3 pulses.
**TIP**: Re-use expensive Neon Tips (≤ 10 times) and replace Neon buffers (as per ref. [22]).
  4.6. Culture transfected cells (as per Step 1) for 4 DIV, and image 50 randomly selected transfected cells (GFP-expressing) per group for neurite growth analysis (as per Step 3).
5. Evaluation of mechanisms of action of neurotrophic strategy in SH-SY5Y cells
  5.1. To investigate, and/or verify, mechanism(s) of action of chosen neurotrophic strategy (pharmacological and/or genetic modifications) in SH-SY5Y cells, perform immunoblotting, immunocytochemistry and real-time PCR experiments (as per ref. [23]).
  5.2. For immunoblotting and real-time PCR experiments, plate 2 ×10^6^ cells/well (in 2 ml of media) in a 6-well plate to maximise yield of protein and RNA extraction.
  5.3. Culture duration should be decided upon depending on protein/mRNA of interest, with hourly time-course experiments recommended to examine signaling pathway activation, and longer time-points recommended to examine changes in expression.
  5.4. Quantify protein levels from immunoblotting and immunocytochemistry experiments by densitometric analysis. For immunoblotting, express levels of protein of interest relative to levels of β-actin loading control. For immunocytochemistry, calculate fluorescence intensity of each individual cell after subtracting background [23,24].

### Evaluation of potential neurotrophic strategies in VM DA neurons

6. Dissection of E14 rat ventral midbrain (VM)
  6.1. To dissect VM, place E14 rat embryos in HBSS in 90 mm Petri dish and keep on ice.
  6.2. Using dissection microscope, make an incision at midbrain-hindbrain boundary, and at forebrain-midbrain boundary, using a dissecting scissors and a curved forceps.
**TIP**: Dissect in lid of petri dish with minimum amount of HBSS to cover lid of dish.
  6.3. Cut dorsal midbrain along midline, and open/flatten-out midbrain to show VM in center. Cut along the lateral border of VM on either side, and then remove meninges.
**TIP**: Put one blade of scissors into the ventricle of midbrain neural tube to cut dorsal midline. Hold meninges down with scissors and carefully peel VM away with forceps.
  6.4. Cut VM tissue cranially and caudally to ensure no fore-/hind-brain tissue is included.
  6.5. Store dissected VMs in 15 ml centrifuge tube in HBSS on ice until completion.
7. Primary cell culture of E14 rat VM
  7.1. Enzymatically dissociate dissected VM tissue by addition of 3 ml of 0.1% trypsin to centrifuge tube, and then incubating at 370C and 5% CO_2_ for 5 min.
  7.2. Add 500 µl of FBS to neutralise the trypsin, and then triturate VM tissue using a pipette tip and a syringe and needle (× 3). Centrifuge VM cells at 1,100 rpm for 5 min.
  7.3. Resuspend cell pellet in 1 ml of Differentiation Media. Prepare a 1:10 dilution of the cell suspension, using the Differentiation Media, for cell counting (as per Step 1).
  7.4. Plate 50 µl of dissociated VM cells, at a density of 50,000 cells/well, in a poly-D-lysine-coated 24-well plate for 2 h at 37°C, before adding 450 µl of media per well.
  7.5. For coating, incubate wells with poly-D-lysine for 20 min, wash (H20 × 3) and dry.
  7.6. Replace half of the Differentiation Media with fresh media every 2–3 DIV.
8. Evaluation of neurite growth-induction ability of potential neurotrophic agent in E14 rat VM DA neurons
  8.1. From 1 DIV, treat E14 VM cells with neurotrophic agent (From Step 1) daily for 4 DIV.
  8.2. At 4 DIV, fix (ice-cold methanol), block (5% BSA) and immunocytochemically stain E14 VM cells for TH/DAPI (as per ref. [23]) to allow visualization of VM DA neurons.
  8.3. Measure total neurite length of 50 randomly selected TH-immunostained neurons per group at 100 × magnification (using a 10 × objective lens) (as per Step 3). For primary neurons, count number of neurite branch points (point a neurite splits into two) also.
**NOTE**: T of 50 µm is recommended for cultured primary neurons.
9. Evaluation of neurite growth-induction ability of genetic modification in E14 rat VM DA neurons
  9.1. For transfection, electroporate E14 VM cells with neurotrophic effect-regulating genetic modification (from Step 1) using the NeonTM Transfection System (as per Step 4).
  9.2. Prepare cell suspension (as per Step 7), and then proceed as per Step 4 until electroporation of the VM cells using the optimized parameters: 1,100 V, 30 ms width and 2 pulses.
  9.3. Culture transfected cells (as per Step 7) for 4 DIV, and image 50 randomly selected transfected VM neurons (GFP-expressing) per group for growth analysis (as per Step 8).
  9.4. For neurite growth analysis of electroporated E14 VM DA neurons, immunocytochemically stain transfected VM cells for TH. Analyze 25 (low abundance) random GFP-expressing, TH-immunostained VM DA neurons per group.
**NOTE**: Anti-GFP antibody may be required if GFP signal is quenched upon fixation.
10. Evaluation of mechanisms of action of neurotrophic strategy in E14 VM cells
  10.1. Investigate, and/or verify, mechanism(s) of action of chosen neurotrophic strategy in E14 VM cells as per Step 5. Plate 2×10^6^ cells/well (in 2 ml of media) in a 6-well plate.
  10.2. For immunocytochemistry experiments, immunocytochemically stain VM cells for both protein of interest and TH, to allow densitometric analysis in VM DA neurons.

### Evaluation of potential neurotrophic strategies in sympathetic neurons

**Figure 1.**
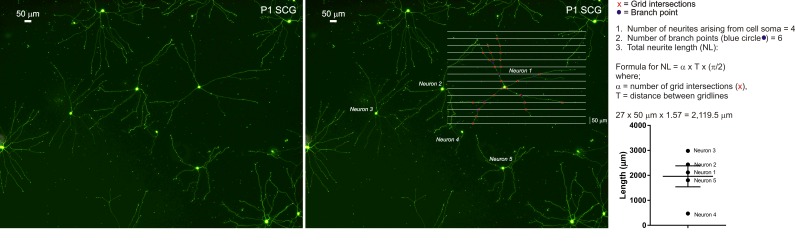
**Neurite growth analysis of SCG sympathetic neurons.** Representative photomicrographs of GFP-expressing P1 rat SCG neurons at 24 h, with gridlines superimposed on ‘Neuron 1’. The grid intersections and branch points are indicated. The neurite length formula is described and an example of neurite growth analysis is depicted. Scale bar = 50 μm.

11. Dissection of P1 mouse superior cervical ganglia (SCG)
  11.1. To dissect SCG, place P1 mouse or rat heads in HBSS in 90 mm Petri dish on ice.
  11.2. Using dissection microscope, remove top of the skull along with the underlying brain.
  11.3. Using the scissors, cut the head sagitally along the midline of the base of the skull.
  11.4. Excise occipital bone to expose the SCG underneath, in each hemi-section of head.
  11.5. Carefully remove SCG with a tweezer, before trimming it of non-SCG tissue.
  11.6. Transfer dissected SCG, using an unplugged flame-polished Pasteur pipette, into a 15 ml conical centrifuge tube, and keep in HBSS on ice until dissections are complete.
**NOTE**: ‘Sticky’ SCG can become attached/stuck to the inside of plastic tips/tubes.
12. Primary cell culture of P1 mouse SCG
  12.1. Chemically dissociate dissected SCG in 1 ml of 0.2% trypsin for 20 min at 370C.
  12.2. Add 500 µl of FBS to neutralise trypsin, and aspirate trypsin/FBS solution carefully.
  12.3. Resuspend SCG in required amount of SCG Media (80 µl per well). Using a narrow pipette tip, forcefully pipette SCG against bottom of 15 ml tube to dissociate cells.
  12.4. Plate 80 µl of SCG cells at a very low density (~200 neurons per dish) in poly-ornithine and laminin-coated 4-well 35 mm tissue culture dishes for 2 h at 370C.
  12.5. For coating, incubate wells with poly-ornithine for 2 h, wash (H20 × 3), and then incubate wells with laminin for 2 h at 370C. Remove laminin and air-dry wells.
  12.6. Add 2 ml of SCG Media to dish, and culture for 24 h at 370C in 5% CO_2_.
13. Evaluation of neurite growth-induction ability of potential neurotrophic agent in P1 SCG sympathetic neurons
  13.1. After 2 h of SCG cell plating, add treatment of neurotrophic agent (from Step 1) to the 2 ml of SCG media for 24 h. Each treatment requires a culture dish with 3 wells of SCG.
**NOTE**: NGF can be excluded from treatment media, and substituted with Boc_D_FMK. NGF should be included for 2 h of plating of SCG cells in wells of dish.
  13.2. At 24 h, fix (ice-cold methanol), block (5% BSA) and immunocytochemically stain SCG cells for TH/βIII-tubulin (as per ref. [23]) to allow visualization of SCG neurons.
**NOTE**: Alternatively, calcein-AM (1:1000) can be added to medium for visualization.
  13.3. Measure total neurite length of 50 randomly selected TH-immunostained neurons per group at 100 × magnification (as per Step 3). Count number of branch points (**Fig. 1**).
14. Evaluation of neurite growth-induction ability of genetic modification in P1 SCG sympathetic neurons
  14.1. For transfection, electroporate SCG cells with neurotrophic effect-regulating genetic modification (from Step 1) using the Neon^TM^ Transfection System (as per Step 4).
  14.2. Prepare cell suspension (as per Step 12), and then proceed to electroporate as per Step 9.
  14.3. Culture transfected cells (as per Step 12) for 24 h, and image 50 randomly selected transfected VM neurons (GFP-expressing) per group for growth analysis (as per Step 13).
15. Evaluation of mechanisms of action of neurotrophic strategy in P1 SCG sympathetic neurons. Investigate, and/or verify, mechanism(s) of action of chosen neurotrophic strategy in SCG cells as per Step 5. Due to low cell numbers, immunocytochemistry experiments are most suitable for examining protein(s) of interest.

### Statistical analyses

16. Using GraphPad Prism software, perform unpaired student’s *t*-test or one-way ANOVA, with a *post hoc* Tukey’s or Bonferroni’s test as required, to determine significant differences (*P* < 0.05) between groups.
17.. Express results as means with standard errors, and repeat each experiment 3 times and in technical triplicate.

## ANTICIPATED RESULTS

### Evaluation of potential neurotrophic strategies in SH-SY5Y cells

**Figure 2.**
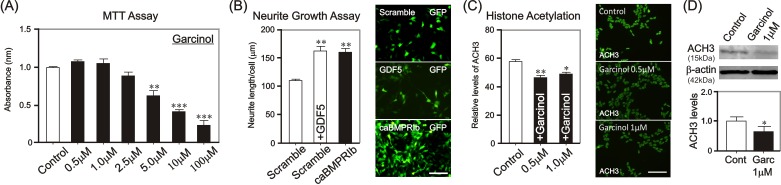
**Evaluation of potential neurotrophic strategies in SH-SY5Y cells. A.** Standardised MTT assay of Garcinol-treated SH-SY5Y cells at 4 DIV. **B.** Graph showing the total neurite length/cell and representative photomicrographs of GFP-expressing GDF5-treated or caBMPRIb-transfected SH-SY5Y cells, compared to an untreated scramble-transfected control at 4 DIV. **C.** Graph showing relative immunofluorescence intensity and representative photomicrographs of ACH3 in SH-SY5Y cells treated for 24 h with either control or Garcinol. As indicated (**P < 0.01, ***P < 0.001 vs. control; One-way ANOVA with post-hoc Tukey’s test). Number of repetitions (N) = 3. Scale bar = 100 μm. **D.** Western blotting showing ACH3 protein levels in SH-SY5Y cells treated for 24 h with control or Garcinol. β-actin was used as a loading control. Graph showing quantification of ACH3 protein levels in relation to β-actin levels, as indicated (*P < 0.05 vs. control; Unpaired student t-test; N = 3).

The authors have previously evaluated potential neurotrophic strategies in SH-SY5Y cells using the procedures detailed above [20,24,25]. Identification of viable working concentrations of potential neurotrophic agents in SH-SY5Y cells can be achieved through MTT Assays. For example, to identify a viable working concentration of the potent inhibitor of histone acetyltransferases (HATs) p300 and PCAF Garcinol (Enzo Life Sciences), SH-SY5Y cells were treated with concentrations ranging from 0.5–100 μM (**Fig. 2A**). These concentrations were selected based on IC50 values of Garcinol being 7 μM and 5 μM for p300 and PCAF, respectively. MTT Assays revealed that Garcinol had no effect on cell viability at concentrations 0.5 μM, 1 μM and 2.5 μM when compared to control (**Fig. 2A**). However, there was a significant reduction in cell viability at concentrations of 5 μM, 10 μM and 100 μM of Garcinol treatment, compared to control (**Fig. 2A**). 0.5 μM and 1 μM were then chosen as viable working concentrations for further Garcinol experimentation.

To evaluate potential neurotrophic strategies for PD-affected neurons, the neurite growth-induction ability of pharmacological agents (*e.g.*, drugs, compounds and small molecules), signaling proteins (*e.g.*, morphogens) and/or genetic (*e.g.*, gene and mRNA) modifications should first be performed in SH-SY5Y cells. For example, treatment of SH-SY5Y cells with 200 ng/ml of GDF5 (provided by Biopharm GmbH), a potent neurotrophic factor for VM DA neurons [13,14,15], or electroporation of SH-SY5Y cells with a constitutively-active, bone morphogenetic protein receptor Ib (caBMPRIb), results in significant increase in their neurite length at 4 DIV when compared to untreated cells transfected with a scramble control plasmid (**Fig. 2B** and ref. [20]).

Following identification of a potential neurotrophic strategy, it is important to evaluate and/or verify its mechanism(s) of action in SH-SY5Y cells, before progressing with the neurotrophic strategy in VM DA neurons and sympathetic neurons. Indeed, Smad signaling has previously been shown to mediate the neurotrophic effects of GDF5 using these methods in SH-SY5Y cells [20]. For example, Garcinol is a p300/PCAF HATs inhibitor, and should thus decrease histone acetylation in SH-SY5Y cells following treatment. To assess the ability of Garcinol to inhibit p300/PCAF HATs, the levels of acetylated-histone H3 were measured in SH-SY5Y cells by immunoblotting and immunocytochemistry, by using a rabbit anti-p-acetylated-histone H3 (ACH3; 1:200; Santa Cruz) primary antibody. Treatment of SH-SY5Y cells with 0.5 μM or 1 μM of Garcinol for 24 h significantly decreased the levels of ACH3 relative to control (**Fig. 2C** and **2D**), following densitometric quantification of ACH3 immunocytochemically-stained SH-SY5Y cells (**Fig. 2C**), and of ACH3 protein levels relative to β-actin loading control (**Fig. 2D**). Therefore, Garcinol induces hypoacetylation of histone H3 in SH-SY5Y cells, likely through inhibition of p300/PCAF HATs.

**Figure 3.**
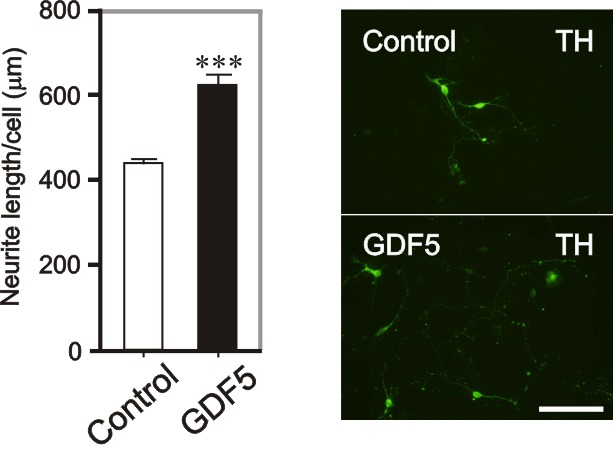
**Evaluation of potential neurotrophic strategies in VM DA neurons.** Graph showing the total neurite length/cell and representative photomicrographs of TH-immunostained E14 VM DA neurons treated with control or GDF5 at 4 DIV. As indicated (***P < 0.001 vs. control; Unpaired student t-test; N = 3). Scale bar = 100 μm.

### Evaluation of potential neurotrophic strategies in dopaminergic neurons and sympathetic neurons

Following evaluation of a potential neurotrophic strategy in SH-SY5Y cells, the next step is to progress with the neurotrophic strategy in the PD-related VM DA neurons and sympathetic neurons. The authors have previously published such evaluations using the procedures outlined above [23,25,26]. For example, treatment of E14 VM cell cultures with GDF5, an effective neurotrophic strategy in SH-SY5Y cells, significantly increased the neurite length of E14 rat VM DA neurons at 4 DIV, when compared to untreated cells (Fig. 3 and ref. [23]). GDF5 treatment also induces significant neurite growth in P1 SCG neurons [26].

The mechanisms of neurotrophic action characterized in SH-SY5Y cells should also be examined in E14 VM DA neurons and P1 SCG sympathetic neurons. We have previously shown that the neurite growth-promoting mechanism of GDF5 is dependent on Smad signaling in SH-SY5Y cells, E14 VM DA neurons and P1 SCG sympathetic neurons [20,23,26]. These findings were determined by genetic manipulations of the Smad signaling pathway following GDF5 treatment, and such experiments can be carried out using the protocol described herein. Furthermore, these studies demonstrate that neurotrophic signaling mechanisms can be conserved between these three cell culture models of the human neurons affected by PD. However, alternative cell-type specific mechanisms should also be considered when assessing potential neurotrophic action mechanisms in VM DA neurons and sympathetic neurons, respectively. For example, sympathetic neuronal growth is largely dependent on NGF signaling, while that of VM DA neurons is not [27,28,29,30].

## TROUBLESHOOTING

Commonly and occasionally encountered problems in the procedure are listed in Table 1, along with the causes of such problems, as well as suggestions to prevent/correct these issues:

**Table 1:** Troubleshooting of procedure.

Step	Problem	Cause	Suggestion
1.2.	Failure of trypsinisation	Inhibitory FBS in residual media	Ensure entire media is washed away with HBSS before trypsin
2.1.	DMSO pools at centre of well & is toxic to the cells located at center	Dense DMSO sits at center of well when added directly to well	Add DMSO(-solubilized drugs) to media in tube, mix and add media/DMSO mixture to well
2.5.	Loss of formazan	Insoluble formazan crystals lost in MTT	Do not agitate plate or disturb formazan when removing MTT
3.3./13.2.	Hyper-exposed calcein-AM staining	Incubation of live cells with calcein-AM > 2 h	Ensure calcein-AM stained cells are imaged in < 2 h
3.5.	Underestimation of neurite length	Gridlines with too large distance between lines	T of 25 µm is recommended for SH-SY5Y cell neurite analysis
4./9./14.	Poor cell survival following transfection	Excessive time taken for electroporation protocol	Perform electroporation protocol in > 30 min
4./9./14.	Poor transfection efficiency & survival	Transfection protocol not optimized	See ‘Transfection of Cultured Neural Cells Optimization’
5./10.	Low protein yield	Excessive lysate dilution	Use 150 µl lysis buffer per well
5./10.	Absence of activated signaling pathway(s)	Phosphorylation of proteins lost	Use phosphatase inhibitors in lysis buffer
6.3.	Meninges on VM tissue	Inefficient removal of meninges from VM	Peel VM tissue from meninges. Do not attempt to cut meninges
7.4.	Unanalysable/misplaced neurons at edge of well	50 µl of plated cells spills to edges of well	Ensure 50 µl of plated cells remains at center of well
8.3./13.3.	Underestimation of neurite length	Gridlines with too large distance between lines	T of 50 µm is recommended for primary neuron growth analysis
9.4.	GFP-positive cells lost	Fixing can quench GFP	Use anti-GFP antibody
11.5.	Wrong ganglia dissected for cell culture	SCG & nodose ganglia similar & close together	SCG is distinctly larger than nodose ganglion
11.6.	Loss of dissected SCG	‘Sticky’ SCG stuck in pipettes &/or tubes	Use unplugged Pasteur pipette that can wash out with trypsin
3./4./8./9./13./14.	Incorrect visualization of target neurons	Inappropriate labeling/identification of neurons	See ‘Visualization of Cultured Neural Cells’

### Transfection of cultured neural cells optimization

**Figure 4.**
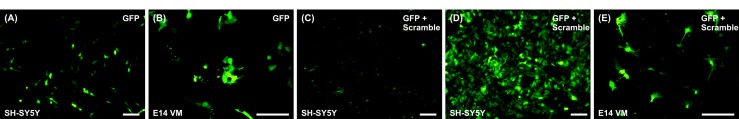
**Transfection of cultured neural cells optimization. A-E.** Representative photomicrographs of SH-SY5Y (A, C, D) or E14 VM cells (B, E) lipo-transfected (A-C) or electroporated (D-E) with GFP (A-E) and/or scramble plasmids (C-E) at 1 DIV post-transfection. Scale bar = 100 μm.

For optimization of lipo-transfection using Transit 2020 Transfection Reagent, 100,000 cells/well of SH-SY5Y and E14 rat VM cells were incubated in 24-well plates at 37^°^C for 1 DIV. Lipo-transfection mixtures containing GFP plasmid at concentrations ranging from 0.25–2 µg/ml and T2020 volumes (room temperature and gently vortexed) ranging from 0.5–4 µl were applied (drop-wise to different areas of their designated wells) to these cultured cells. It was identified that 0.5 µg of GFP plasmid and 0.5 µl of T2020 gives the highest transfection efficiency (~20–30%) in SH-SY5Y cells (**Fig. 4A**), while 1.5 µg of GFP plasmid and 1 µl of T2020 gives the highest transfection efficiency (10–20%) in E14 VM cells (**Fig. 4B**). Similar transfection efficiencies but reductions in cell viability were observed with higher volumes (2–4 µl) of T2020 (data not shown). For optimization of co-lipo-transfection, 0.5 µg of the GFP plasmid and 1 µg of scramble vector were mixed with T2020 volumes ranging from 0.25–2 µl in SH-SY5Y cells. This identified that co-lipotransfection of SH-SY5Y cells with GFP and scramble plasmids and 2 µl of T2020 gives the highest co-transfection efficiency (~10–20%) in SH-SY5Y cells (**Fig. 4C**). However, there was a decrease in transfection efficiency and GFP expression in co-transfected SHSY5Y cells, when compared to GFP-transfected cells (**Fig. 4A** and **4C**). Co-lipo-transfection of E14 VM cells with GFP and scramble plasmids using T2020 volumes ranging from 0.25–2 µl resulted in no successfully co-lipo-transfected E14 VM cells (data not shown).

As a result of the poor co-transfection efficiencies using lipo-transfection, optimization of the electroporation of SH-SY5Y and E14 VM cells was carried out. Cells were electroporated with GFP and scramble plasmids using a range of parameters: voltage (500–1500 V), width (10–30 ms) and pulses (1–3). Optimal parameters for SH-SY5Y cell electroporation were found to be 1200 V, 20 ms width and 3 pulses, which resulted in a co-transfection efficiency of ~70–80% (Fig. 4D), which was higher than that achieved by lipo-transfection (**Fig. 4A** and **4C**). Co-transfection of E14 rat VM cells with GFP and scramble plasmids by electroporation was also performed successfully, with the highest efficiency (30–40%) achieved using the following parameters; 1100 V, 30 ms width and 2 pulses (Fig. 4E). Due to cell type heterogeneity within primary VM cultures, the proportion of transfected VM neurons which are TH-positive is ~30%.

### Visualization of cultured neural cells

**Figure 5.**
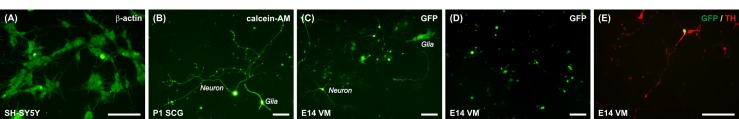
**Visualization of cultured neural cells. A-E.** Representative photomicrographs of SH-SY5Y (A), P1 SCG (B) or E14 VM (C-E) cells immunocytochemically stained for β-actin (A) or TH (E), visualized with calcein-AM (B), and/or transfected with GFP (C-E) at 1 (B) or 4 (A, C-E) DIV. Neurons and Glia are indicated in B and C. Scale bar = 100 μm.

To visualize cultured cells for neurite growth analysis, immunocytochemically stain SH-SY5Y cells, VM DA neurons and SCG sympathetic neurons for β-actin (**Fig. 5A**), TH (**Fig. 3**) and TH/βIII-tubulin, respectively. Alternatively, SH-SY5Y cells and SCG sympathetic neurons can be visualized using calcein-AM (**Fig. 5B**). However, VM cultures contain a mixed population of neuronal subtypes, and calcein-AM is therefore unsuitable for selective visualization of VM DA neurons. When analyzing GFP-positive and/or calcein-AM-positive cells in primary cultures of VM (**Fig. 5C**) and SCG (**Fig. 1** and **Fig. 5B**), ensure that only neurons, and not glia, are analyzed. Due to the sensitive nature of primary neuronal cultures, ensure that unhealthy cultures are not included in any analyses (**Fig. 5D**). Finally, to selectively analyze VM DA neurons in electroporated VM cell cultures, immunocytochemically stain for TH (and GFP if required), and then analyze neurite growth of GFP- and TH-positive VM neurons (**Fig. 5E**).

## CONCLUSIONS

In order to develop novel NTF therapeutic strategies for PD, we need a systematic protocol for pre-clinical evaluation of potential neurotrophic strategies in the neuronal populations that are affected by PD. Before proceeding to experimentation in PD animal models, we first need to identify and evaluate potential neurotrophic agents *in vitro*. The present *in vitro* protocol allows a step-wise evaluation of potential neurotrophic strategies first in SH-SY5Y cells, and then in VM DA neurons and sympathetic neurons, populations which degenerate in PD [2-6,8-10]. As neurotrophic factors function to promote the survival and neurite growth of neurons during development and in adulthood, this protocol focuses on the induction of neurite growth as the primary measure of neurotrophic action. Indeed, neuro-protection/-restoration of PD-affected neurites is thought to be an appropriate target for effective therapeutic intervention in PD [19], and thus neurotrophic strategies which are effective at promoting neurite growth, identified using this protocol, have direct clinical potential in PD. In addition to this, the neurite growth methods described in this protocol can be applied to the study of neuronal growth in other scientific contexts, such as the study of regulators of neuronal development, innervation and plasticity. Similarly, the detailed description of primary culture of VM DA neurons and sympathetic neurons permits any desired study with these cells. For example, the VM DA neuronal cultures herein can also be used in the study of psychiatric disorders, in which a subset of these neurons are also implicated [31-33].

It has been previously suggested that NTF therapy clinical trials may have failed due to a disruption of the neurotrophic signaling mechanism in the PD brain. Therefore, the mechanisms of action of neurotrophic strategies need to be characterized and validated in pre-clinical studies. The protocol described in this paper permits the evaluation of the mechanisms of action of the neurtrophic strategy under investigation. Thereafter, such neurotrophic action mechanism(s) should be demonstrated as functional in rodent models of PD (including α-synuclein rat model of PD) and the PD brain, before clinical trials with the potential neurotrophic strategy are considered in PD patients. Finally, the invasive nature and physiological limitations of intracerebral delivery of NTFs to the PD brain limits the potential of this therapy [15]. Novel neurotrophic strategies that could be applied peripherally, and which could protect the degenerating neurons in both the central and peripheral nervous system, would be the most suitable NTF therapy for PD. Evaluation of potential neurotrophic agents, such as blood-brain-barrier-permeable small molecules, which ideally could be modified to target the neuronal populations affected by PD (*e.g.*, selective uptake by TH-positive neurons) using our protocol would facilitate the development of such a therapy. In summary, this *in vitro* protocol provides a reliable, reproducible, robust and cost-effective means to identify, evaluate and characterize promising neurotrophic strategies in PD-related neurons, and serves as a screening process to select novel therapeutic approaches for PD which are suitable for further testing in animal models and the clinic.

## Abbreviations used

α: number of times the neurites intersect the grid lines
ACH3: p-acetylated-histone H3
BSA: bovine serum albumin
caBMPRIb: constitutively-active bone morphogenetic protein receptor Ib
DA: dopaminergic/dopamine
DAPI: 4’-6-diamidino-2-phenylindole
DMEM-F12: Dulbecco’s Modified Eagle Medium Nutrient Mixture F-12
DMSO: dimethyl sulfoxide
DIV: day(s) in vitro
E: embryonic day
FBS: foetal bovine serum
GDF5: growth/differentiation factor 5
GDNF: glial cell line-derived neurotrophic factor
GFP: green fluorescent protein
HAT: histone acetyltransferase
MTT: Thiazolyl Blue Tetrazolium Bromide
N: number of repetitions
NGF: nerve growth factor
NTF: neurotrophic factor
NTN: neurturin
P: postnatal day
PFA: paraformaldehyde
PBS: phosphate-buffered saline
PD: Parkinson’s disease
SCG: superior cervical ganglion/ganglia
SDS: sodium dodecyl sulphate
T: distance between the gridlines on the magnified image
TH: tyrosine hydroxylase
VM: ventral midbrain
